# Supplementary material for: Formulation and Evaluation of Loperamide HCl Oro Dispersible Tablets
Source: Pharmaceuticals (Basel). 2020 May 18;13(5):100. doi: 10.3390/ph13050100 (PMC7281284; doi:10.3390/ph13050100)

Supplementary data for the following article:

Article

FORMULATION AND EVALUATION OF LOPERAMIDE HCl ORO DISPERSIBLE TABLETS

Blasco Alejandro, Torrado Guillermo and Peña M Ángeles

Figure S1. DSC study of API (loperamide HCl) and excipients (mannitol, HPMC, anise extract, Explotab®, magnesium stearate, Emcompress®, menthol and sodium cyclamate).

Figure S2. IR of loperamide HCl and the excipients selected in KBr in the composition of the ODT designed.

Figure S3. (A) Loperamide HCl SEM image and the excipients selected in the composition of the ODT designed.

.

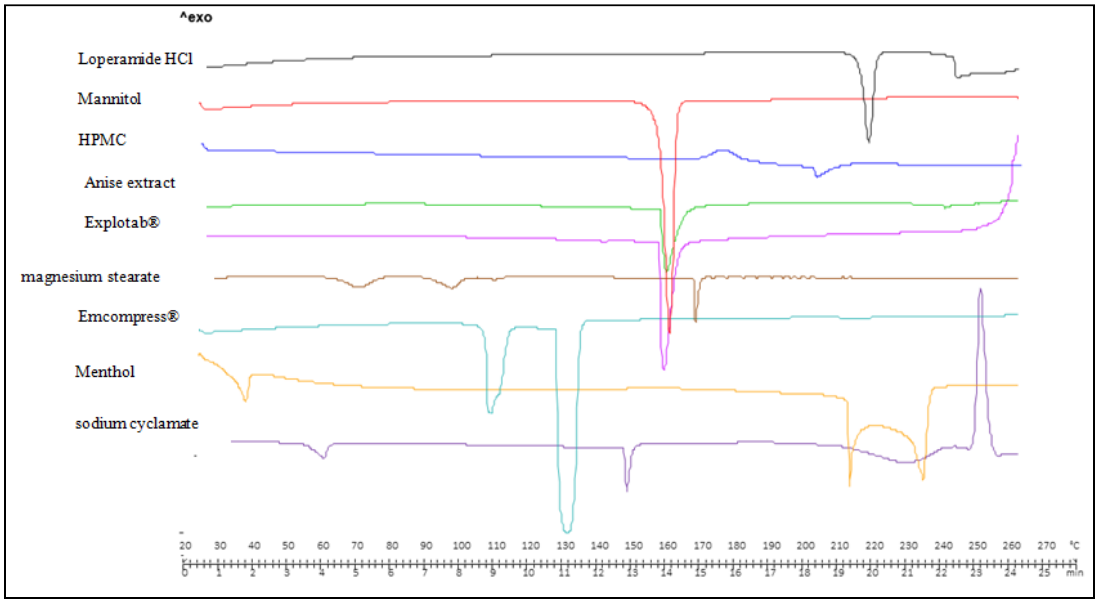
^exo
Loperamide HCl
Mannitol
HPMC
Anise extract
Explotab®
magnesium stearate
Emcompress®
Menthol
sodium cyclamate
20 30 40 50 60 70 80 90 100 110 120 130 140 150 160 170 180 190 200 210 220 230 240 250 260 270 °C
0 1 2 3 4 5 6 7 8 9 10 11 12 13 14 15 16 17 18 19 20 21 22 23 24 25 min

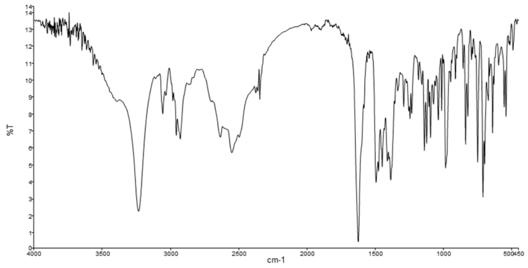

Figure5A. Loperamide HCl

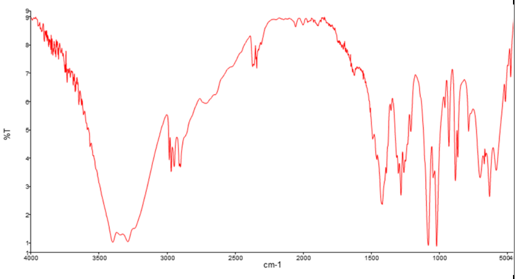

Figure 5B. Mannitol

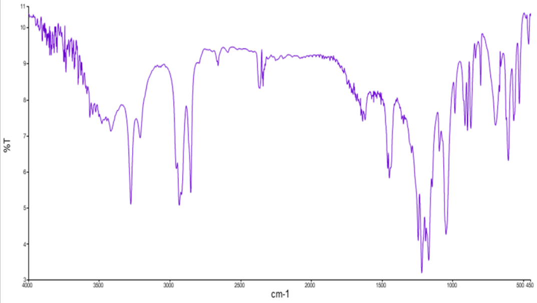

Figure 5C. Sodium cyclamate

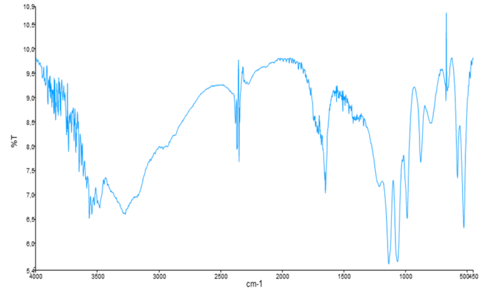

Figure 5D. Calcium hydrogen phosphate dihydrate

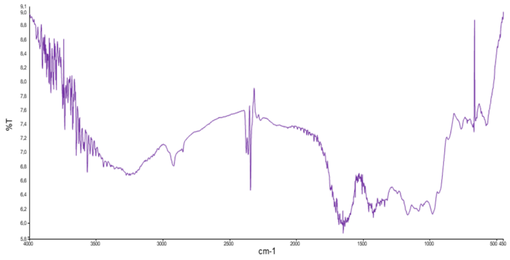

Figure 5E. Sodium starch glycolate (type A)

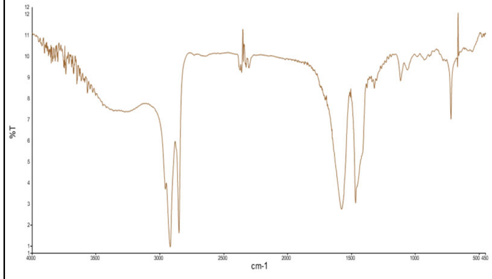

Figure5F.Magnesium stearate

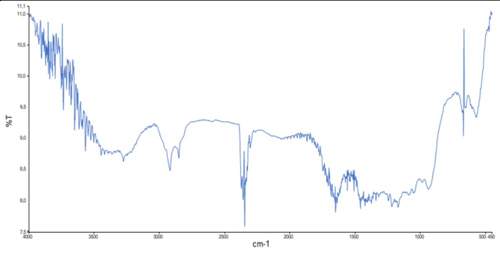

Figure 5G. Hypromellose

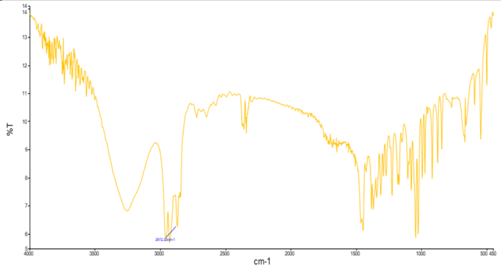

Figure 5H. Menthol

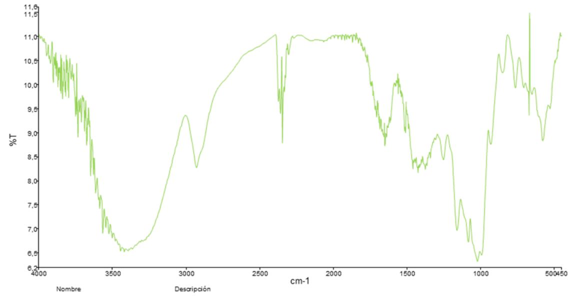

Figure 5I. Anise extract

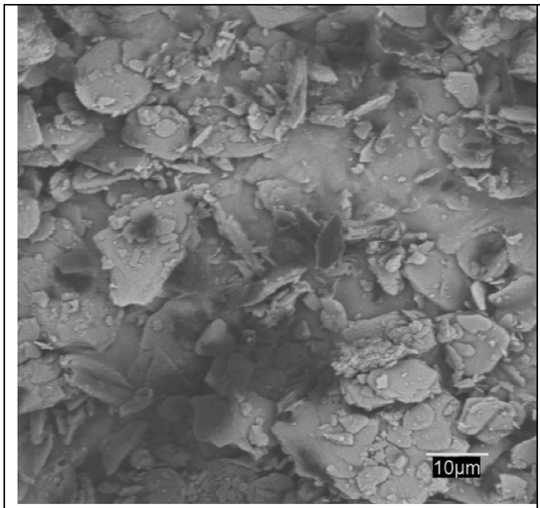

Loperamide HCl

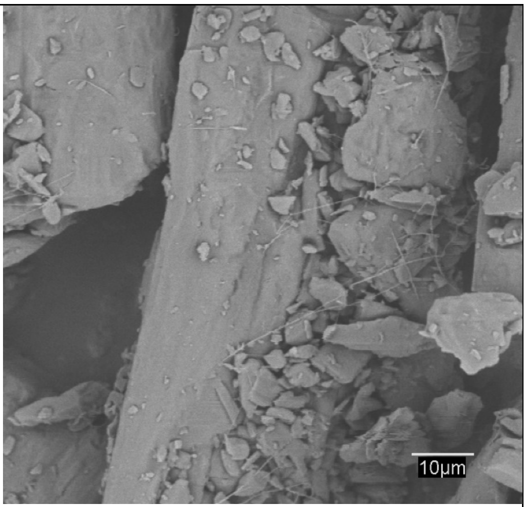

Mannitol

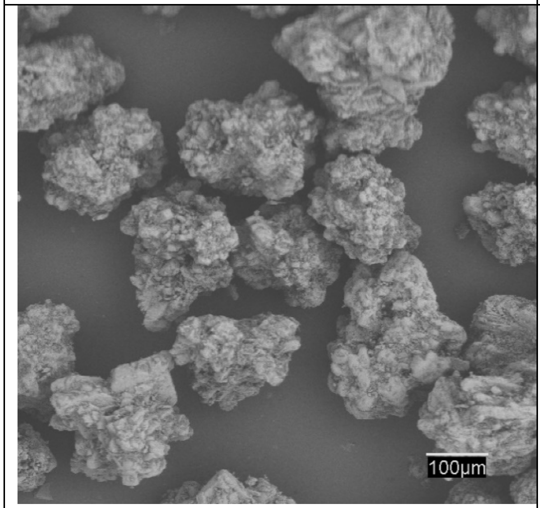

Calcium hydrogen phosphate dihyhidrate

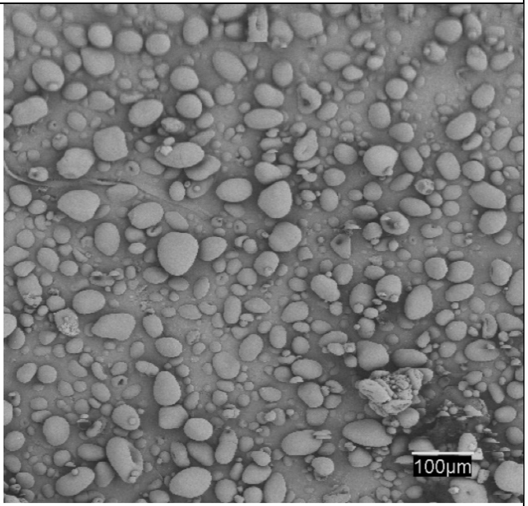

Sodium starch glycolate type A

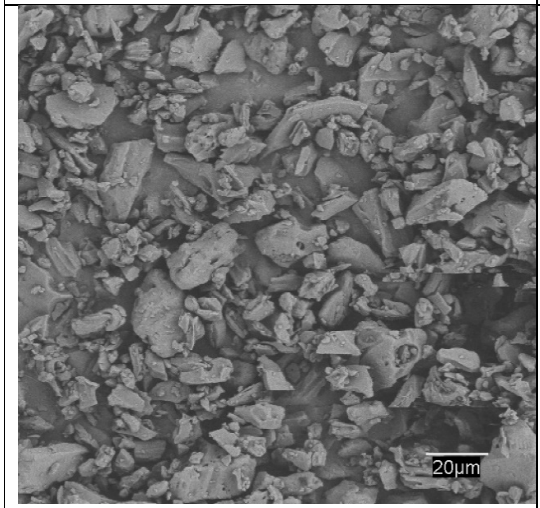

Magnesium stearate

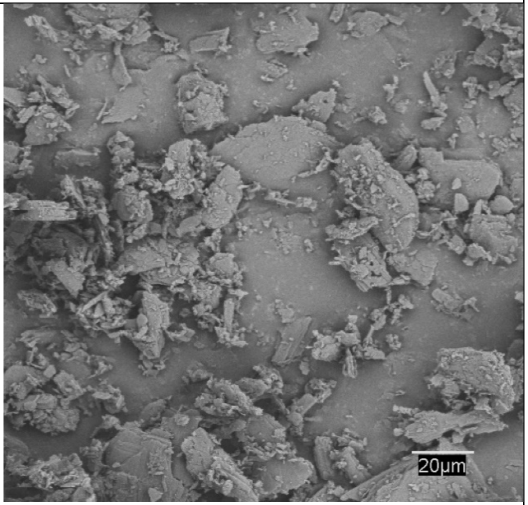

Sodium cyclamate

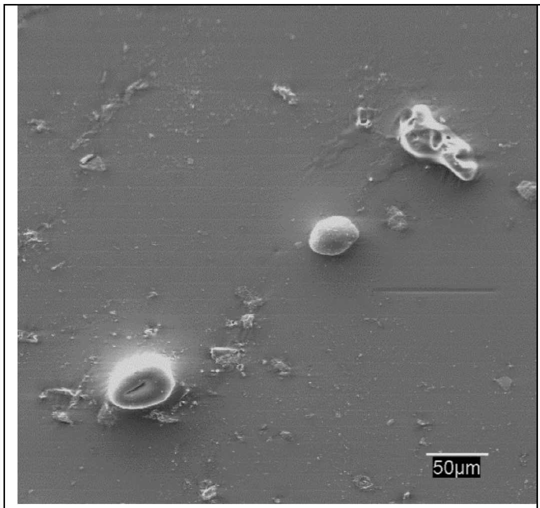

Menthol

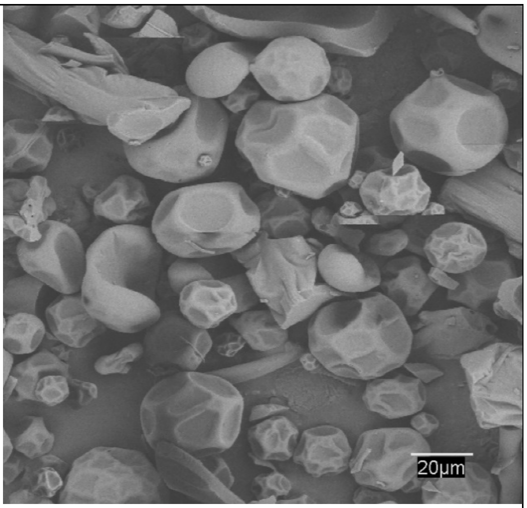

HPMC

Anise extract

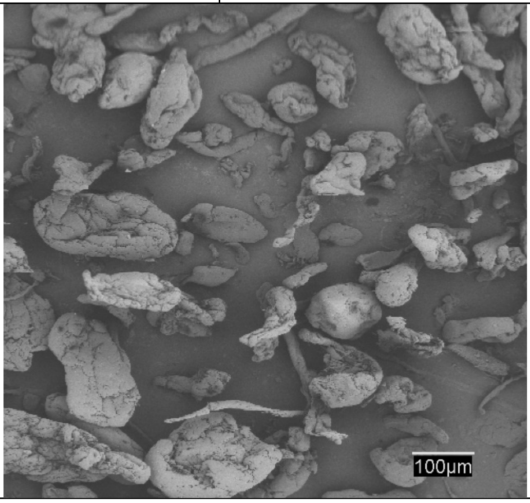

Supplement: Supplementary file 1 [file pharmaceuticals-13-00100-s001.pdf]
